# Predicting mortality of patients with acute kidney injury in the ICU using XGBoost model

**DOI:** 10.1371/journal.pone.0246306

**Published:** 2021-02-04

**Authors:** Jialin Liu, Jinfa Wu, Siru Liu, Mengdie Li, Kunchang Hu, Ke Li

**Affiliations:** 1 Information Center, West China Hospital, Sichuan University, Chengdu, Sichuan Province, China; 2 Department of Medical Informatics, West China Medical School, Chengdu, Sichuan Province, China; 3 School of Life Science & Technology, University of Electronic Science & Technology of China, Chengdu, Sichuan Province, China; 4 Department of Biomedical Informatics, University of Utah, Salt Lake City, UT, United States of America; BronxCare Health System, Affiliated with Icahn School of Medicine at Mount Sinai, NY, USA, UNITED STATES

## Abstract

**Purpose:**

The goal of this study **is** to construct a mortality prediction model using the XGBoot (eXtreme Gradient Boosting) decision tree model for AKI (acute kidney injury) patients in the ICU (intensive care unit), and to compare its performance with that of three other machine learning models.

**Methods:**

We used the eICU Collaborative Research Database (eICU-CRD) for model development and performance comparison. The prediction performance of the XGBoot model was compared with the other three machine learning models. These models included LR (logistic regression), SVM (support vector machines), and RF (random forest). In the model comparison, the AUROC (area under receiver operating curve), accuracy, precision, recall, and F1 score were used to evaluate the predictive performance of each model.

**Results:**

A total of 7548 AKI patients were analyzed in this study. The overall in-hospital mortality of AKI patients was 16.35%. The best performing algorithm in this study was XGBoost with the highest AUROC (0.796, p < 0.01), F1(0.922, p < 0.01) and accuracy (0.860). The precision (0.860) and recall (0.994) of the XGBoost model rank second among the four models.

**Conclusion:**

XGBoot model had obvious advantages of performance compared to the other machine learning models. This will be helpful for risk identification and early intervention for AKI patients at risk of death.

## Introduction

Acute kidney injury (AKI) is a common condition with a high mortality rate, morbidity, high cost, and risk of developing chronic kidney disease [1]. It is also a global health issue [2]. Although the level of diagnosis and treatment has improved in recent years, the burden of disease caused by AKI is still very high, especially in the intensive care unit [1]. In clinical practice, the estimation of the mortality risk is helpful for triage and resource allocation, to determine the appropriate level of care, and even to discuss the expected outcomes with patients and their families [3].

In recent years, machine learning has been widely used to predict disease risk. Risk adjustment and mortality prediction are critically important for comparing outcomes across interventions and health systems. For example, Sevag Demirjian et al. (2011) constructed an LR (logistic regression) model to predict mortality in AKI patients and compared it with the prediction results of APACHE II (Acute Physiology and Chronic Health Evaluation II) score, SOFA (Sequential Organ Failure Assessment) score and CCF (Cleveland Clinic Foundation) score [4]. Ke Lin et al. (2019) used the RF (random forest) algorithm to build a mortality prediction model, and predicted the mortality risk of AKI patients in ICU. Their model was compared with SVM (support vector machine), ANN (artificial neural network), and Customized SAPS-II (Simplified Acute Physiology Score-II) scores [5]. These studies showed LR and RF exhibited good discrimination, and remarkable accuracy [4, 5]. Many advanced AI models, such as deep learning techniques, have shown remarkable accuracy in mortality prediction [6, 7]. However, in clinical real-world scenarios, the inability to provide sufficient data for model training has prevented AI models from performing well. The AI models perform poorly when dealing with relatively small datasets and cannot be widely used in clinical practice [8]. While machine learning models have a good predictive performance on smaller datasets. However, a single machine learning approach often leads to overfitting and difficulty in dealing with the large number of unbalanced datasets that occur in actual problems. To compensate for the shortcomings of a single machine learning method, the ensemble learning technique based on the GBDT (gradient boosting decision tree) algorithm was developed and has gradually become the mainstream approach in the field of machine learning research [9, 10]. XGBoost is a highly efficient boosting ensemble learning model that originated in the decision tree model, which uses the tree classifier for better results of prediction and higher operation efficiency [11, 12].

The purpose of this study is to use XGBoost to construct a predictive mortality model for AKI patients in the ICU, and to use the publicly available database eICU Collaborative Research Database V2.0 as a data source [13]. In addition, the performance of the XGBoost model was compared with LR, SVM, and RF model.

## Method

### Dataset

This study used the eICU-CRD V2.0 with 200,859 admissions between 2014 and 2015 at 208 hospitals of the United States (https://eicu-crd.mit.edu/). The database was a multicenter ICU database with a high granularity of data. It included data on patient vital sign measurements, care plan documentation, nurse charting, disease severity measures, laboratory variables, diagnostic information, and treatment information [13].

### Patients

**Fig 1:**
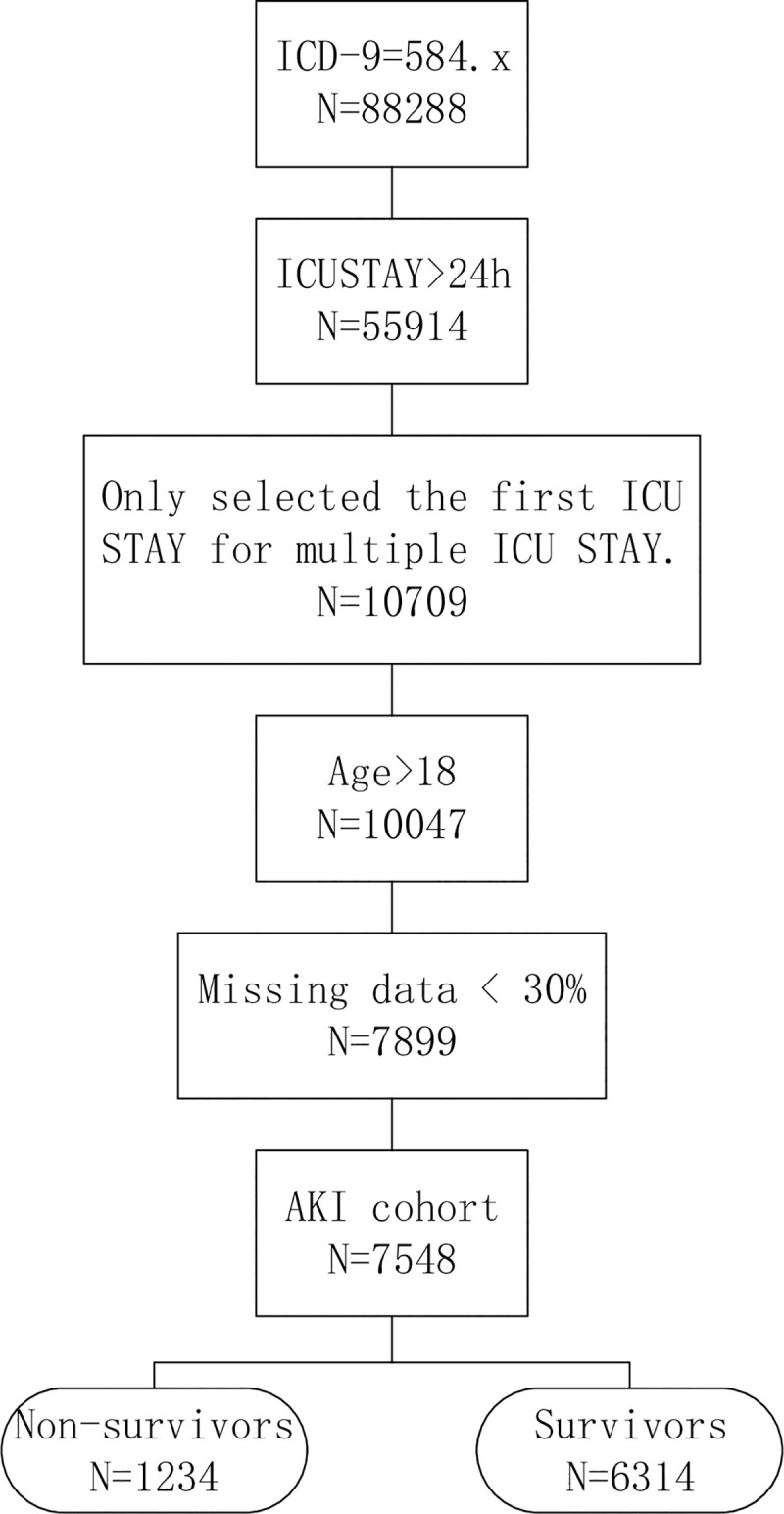
The patients’ selection process. ICD-9: International Classification of Diseases, Ninth Revision.

All patients in eICU-CRD version v 2.0 databases were eligible for inclusion in the present investigation. The following inclusion criteria were used: (1) All AKI patients (ICD-9, 584.x) admitted to the ICU with a length of stay> 24 hours; (2) age 18 years or more; and (3) patients with more than 30% missing values were excluded from the analysis [5]. As for those patients who were admitted to ICU for more than once, only data of the first ICU stay were used. The patients’ selection process was shown in Fig 1.

### Predictor variables

The variables used to predict the mortality of AKI include various demographic, clinical, and laboratory variables. These variables were based on experts’ opinion and roughly matched the variables used in the Acute Physiological and Chronic Health Assessment II (APACHE II) [14]. These variables were collected at admission within the first 24 hours of ICU admission. These variables in the specified period were collected, and in case of missing variables, the mean variable was assigned. After extracting all the characteristic variables, the Lasso (least absolute shrinkage and selection operator) regression method was used to select and filter the variables with the top 25 importance [15, 16].

### Prediction models

To confirm the effectiveness of the XGBoost model in predicting AKI mortality, we used the following widely used machine learning models (LR, SVM, RF) for comparison and summarized the advantages and disadvantages of each of these models (Table 1).

**Table 1:** Advantages and disadvantages of each models.

Models	Advantages	Disadvantages
LR [24]	LR is easier to implement, interpret, very fast calculations at classification and very efficient to train.	When the number of observations is less than the number of features, overfitting may result.
SVM [25, 26]	SVM can be used for linear and non-linear classification and regression problems. It provides a good out-of-sample generalization.	Kernel models can be quite sensitive to over-fitting the model selection criterion.
RF [27]	It can be used for both regression and classification tasks, and no need for feature normalization.	Not interpretable.
		Performance is not good when there is class imbalance.
	Avoids over-fitting.	
XGBoost [28]	Do not require feature engineering (missing values imputation, scaling and normalization).	For numeric features only.
		Leads to overfitting if hyperparameters are not adjusted correctly.
	It can be used for classification, regression or ranking.	
	Extremely fast (parallel computation), and highly efficient.	

LR is a widely used statistical model. It is used to calculate the probability of occurrence of binary events and deal with classification problems. LR allows for multivariate analysis and modeling of a binary dependent variable. The multivariate analysis estimates the coefficients of each predictor included in the final model (e.g., log odds or hazard ratios) and adjusts them based on other predictors in the model. These coefficients quantify the contribution of each predictor to the risk estimate of the outcome [17].

SVM is a supervised machine learning algorithm. It is a binary classification method that separates two classes by a linear boundary and relies on extended linearity. In this algorithm, the main goal is to find the farthest distance between two classes, leading to more accurate classification and a reduction in generalization error [18].

RF is an ensemble algorithm, which combines multiple decorrelated decision tree prediction variables based on each subset of data samples [19]. RF is not only fast, easy to implement, and produces precise predictions, but it can also handle a large number of input variables without overfitting [20].

XGBoost is an improved algorithm based on the gradient boosting decision tree, which can efficiently construct boosted trees and run in parallel. The boosted trees in XGBoost is divided into regression trees and classification trees. The core of the algorithm is to optimize the value of the objective function [21]. XGBoost has the advantages of scalability in all scenarios, and fast [22]. The model works by combining a set of weaker machine learning algorithms to obtain an improved machine learning algorithm as a whole [23].

In model development and comparison, we employed 5-fold cross-validation, which provides a more stable and reliable way to measure the performances of models.

### Model parameter setting

Based on the literature review and our experience, we chose the tuning parameter. For Lasso, we used alpha: ‘0.01’ to select the top 25 important variables. For the LR model, we set penalty: ‘l2’, solver: ‘liblinear’; In the SVM model, we used ‘rbf’ kernel and used ‘auto’ for gamma to train the classifier; For RF model, we set criterion: ‘gini’, and used the default parameter for other model parameters; For XGBoost model, we set learning rate: ‘0.1’, max_depth: ‘3’, objective: ‘binary:logistic’, booster:‘gbtree’, gamma: ‘0’.

### Model evaluation

Each model was evaluated according to precision, recall, accuracy, F1 score, and AUROC (area under the receiver operating characteristic) curve. In this study, accuracy is the ratio of correctly predicted observations to the total number of observations. Precision refers to the ratio of correctly predicted positive observations to the total number of predicted positive observations. The recall is the ratio of correctly predicted positive observations to all actually positive observations. F1 Score is a harmonic mean of precision and recall. AUROC is a probability curve that graphically displays the trade-off between recall and specificity [29, 30].

## Results

### Participant characteristics

A total of 7,548 patients with AKI were included in the final cohort for this study, among which 1,234 (16.35%) died. In the 7,548 AKI patients, the proportion of male sex in the death group (57.7%) was higher than that in the survival group (55.7%). It was statistical significantly (P <0.01). The average age of the non-survival group and surviving group patients was 67.4 (SD ± 14.4) and 65.9 (SD ± 15.5) years, respectively. The non-survival group patients were statistically significantly older than survival group patients (P <0.01). The predominantly white population accounted for 76% of these patients. Patients in the non-survival group (7.5±8.2) had marked longer days of ICU stay than those in the survival group (6.6±9.4), and had statistical difference (P <0.01). Demographics of patients with AKI are shown in Table 2.

**Table 2:** Demographics of patients with AKI.

Variable	Non-survivors (n = 1234)	Survivors (n = 6314)	P
Gender	−	−	−
Female	522(42.3%)	2794(44.3%)	<0,001
Male	712(57.7%)	3520(55.7%)	
Age(year)	67.4±14.4	65.9±15.5	<0.01
Height(cm)	169.4±11.5	169.3±12.3	0.91
Weight(kg)	91.2±28.0	89.7±29.7	0.237
BMI	39.65±11.81	30.58±9.91	0.140
Ethnicity	−	−	−
Caucasian	951(77.1%)	4816(76.%)	0.5477
African-American	120(9.7%)	736(11.7%)	0.0502
Hispanic	72(5.8%)	347(5.5%)	<0.001
Other	91(7.4%)	415(6.6%)	0.3024
ICU days (mean,SD)	7.5±8.2	6.6±9.4	0.003

### Variable selection

**Fig 2:**
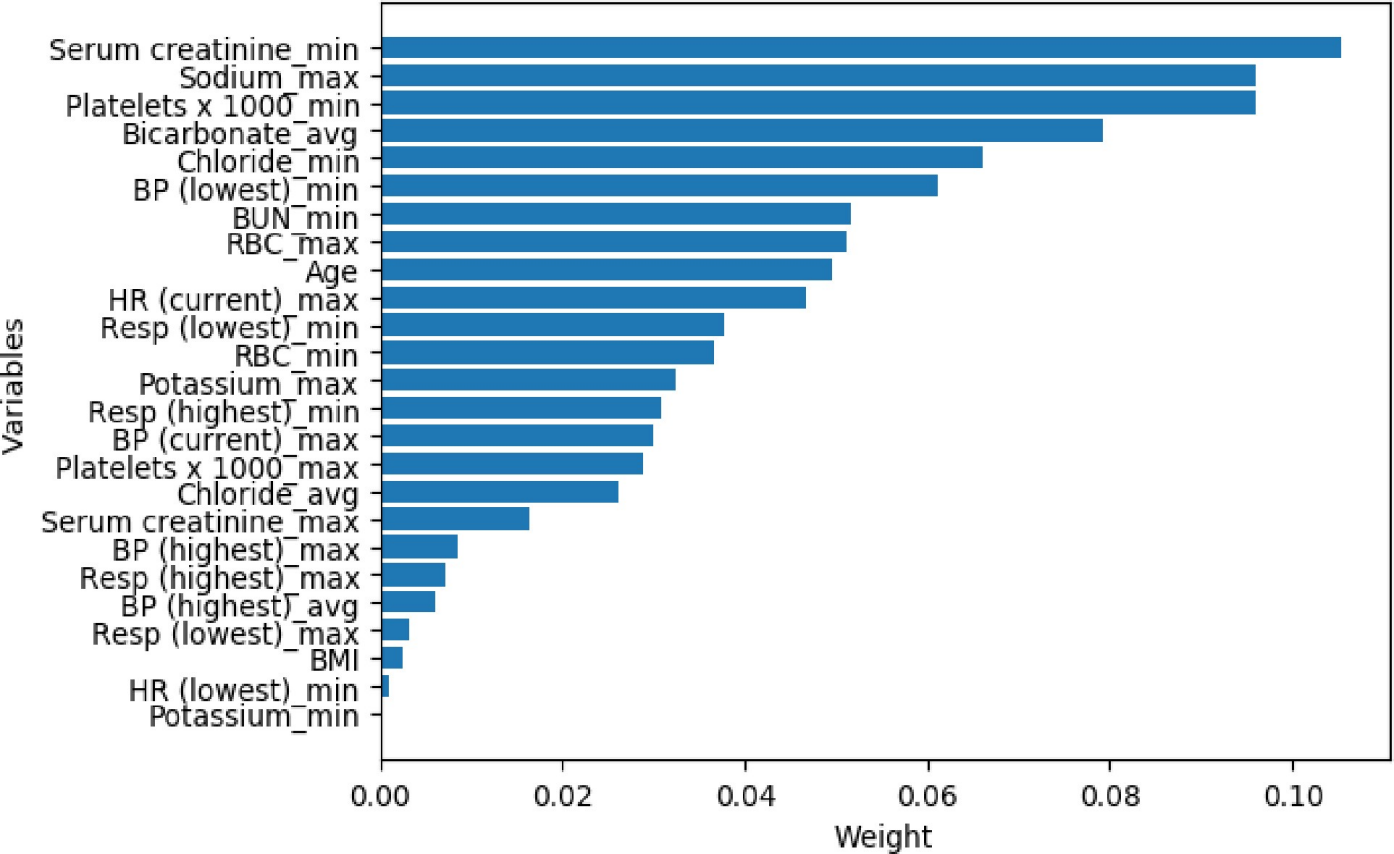
The weights of variables importance. The variables were collected from the eICU-CRD V2.0 database with the AKI patients’ admission date from 2014 to 2015. BP: blood pressure; BUN: blood urea nitrogen; RBC: red blood cell; HR: heart rate; Resp: respiratory rate; min: minimum; max: maximum; avg: average.

To detect the importance of variables in predicting mortality in AKI patients, Lasso (least absolute shrinkage and selection operator) was applied for feature selection. Lasso is a regression analysis method that uses L1 constraint to perform variable selection and regularization, providing a base to select a subset of the available covariates for use in the final model [12]. The Lasso selected the top 25 predictor variables (among 64 total variables) and weight (Fig 2). Creatinine (min) was the most important predictor variables for all prediction horizons, followed very closely by Sodium (max), markers of Platelets, Bicarbonate (average), and Chloride (min) (Tables 2 and 3).

**Table 3:** All predictor variables for non-survivors and survivors.

Variable(SD)	Non-survivors (n = 1234)	Survivors (n = 6314)	P
Serum creatinine min	1.70(1.19)	1.45(1.11)	<0.001
Sodium max	140.46(6.85)	139.62(6.32)	<0.001
Platelets x1000 min	163.39(105.37)	185.67(99.72)	<0.001
Bicarbonate avg	21.21(5.37)	22.35(5.20)	<0.001
Chloride min	104.26(7.85)	104.36(7.70)	<0.001
BP Lowest min	92.91(28.63)	99.50(28.23)	0.926
BUN min	42.47(26.99)	43.13(27.27)	0.066
RBC max	3.65(0.80)	3.60(0.74)	0.002
Age	67.41(14.37)	65.96(15.50)	<0.001
HR Current max	97.42(22.32)	92.55(21.12)	<0.001
Resp Lowest min	18.67(6.17)	17.42(6.05)	<0.001
RBC min	3.38(0.82)	3.44(0.76)	0.411
Potassium max	4.61(0.85)	4.50(0.84)	0.001
Resp Highest min	25.89(7.56)	24.20(7.92)	<0.001
BP Current max	115.37(25.57)	120.12(26.09)	<0.001
Platelets x1000 max	186.75(111.91)	199.44(104.19)	0.002
Chloride avg	105.66(7.64)	105.51(7.50)	0.201
Serum creatinine max	3.42(1.85)	3.17(2.39)	<0.001
BP Highest max	132.38(31.72)	133.32(31.02)	0.416
Resp Highest max	27.40(8.21)	25.57(8.72)	<0.001
BP Highest avg	128.49(30.03)	130.02(29.28)	0.914
Resp Lowest avg	20.01(6.55)	18.56(6.29)	0.002
BMI	39.65(11.81)	30.58(9.91)	0.140
HR Lowest min	85.76(21.53)	83.37(20.57)	0.359
Potassium min	3.99(0.77)	4.02(0.70)	0.032

BP: blood pressure; BUN: blood urea nitrogen; RBC: red blood cell; HR: heart rate; Resp: respiratory rate.

### Model performance

**Fig 3:**
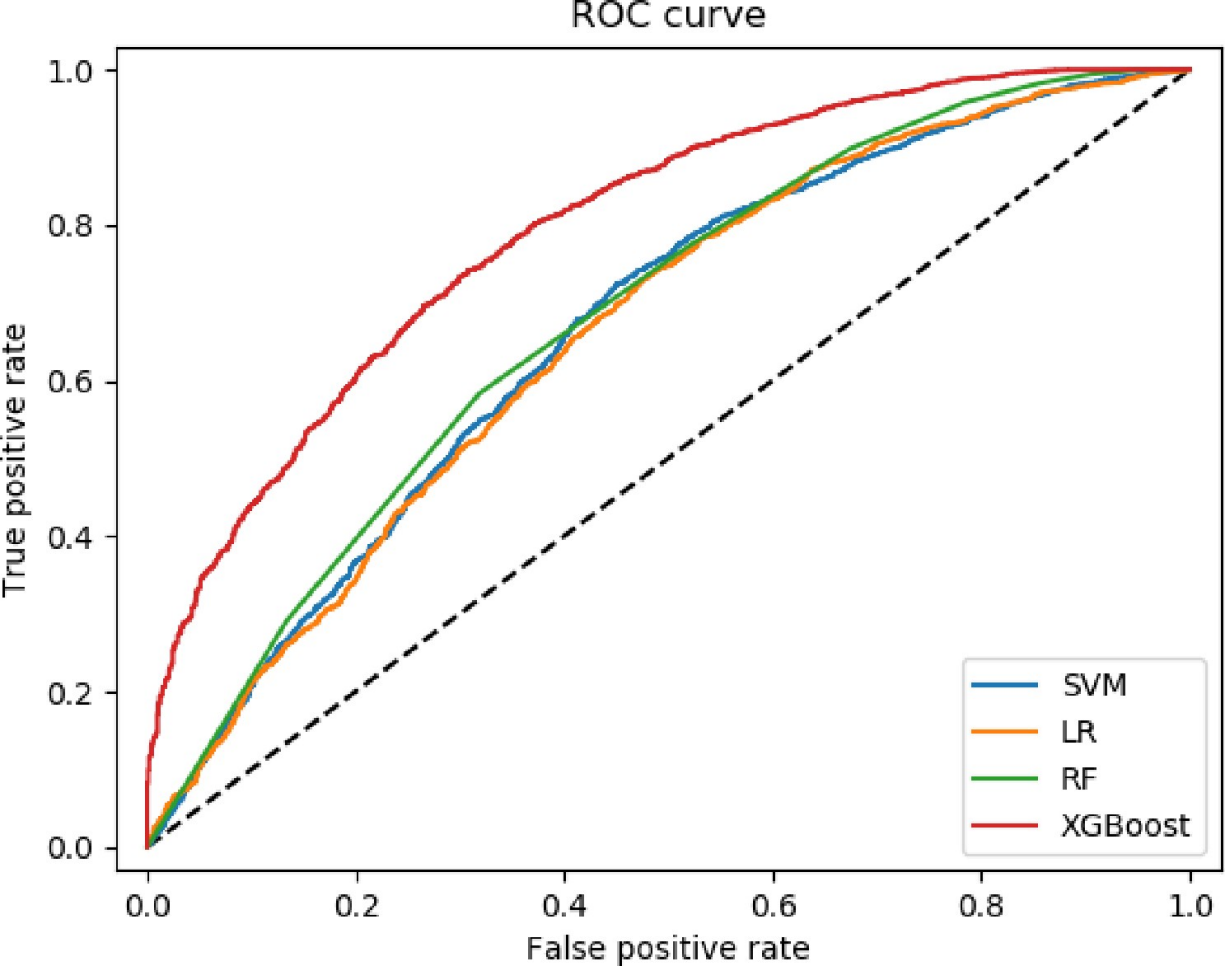
The ROC curve among the four models of AKI patients.

The results in the four machine learning methods found in the 5-fold cross-validation are shown in Table 4. The AUROC (0.796), accuracy (0.860), and F1 score (0.922) of XGBoost were higher than all other models. The precision and recall of the XGBoost model were the second-best among the four models. XGboost was superior to other models in terms of AUROC and F1, and had statistical significance (P<0.01). The lowest F1 score (0.910) and AUROC (0.662) were LR and RF, respectively (Table 4). The AUROC curves of these predictive models were shown in Fig 3.

**Table 4:** Comparison of mortality prediction performance among the four models of AKI patients.

	AUROC	Precision	Recall	Accuracy	F1	P(AUROC)	P(F1 score)
LR	0.662	0.842	0.992	0.837	0.911	-	-
SVM	0.667	0.837	**0.999**	0.837	0.911	<0.01vs.LR	<0.01vs.LR
RF	0.692	**0.862**	0.956	0.836	0.910	<0.01vs.LR	<0.01vs.LR
						<0.01vs.SVM	<0.01vs.SVM
XGBoost	**0.796**	0.860	0.994	**0.860**	**0.922**	<0.01vs. LR	<0.01vs. LR
						<0.01vs SVM	<0.01vs SVM
						<0.01vs RF	<0.01vs RF

## Discussion

This study found a higher proportion of male sex in the AKI non-survival group patients than the survival group patients, and there was a statistical difference between the two groups (P<0.001). Elderly patients (average age 67.4 years old) were associated with an increased risk of death, and there was a statistical difference (P<0.01). Some researchers showed that there was a significant increase in old age and males in deceased AKI patients [31, 32]. At eGFR (estimated glomerular filtration rate) 80 ml/min/1.73 m^2^, the older age itself was linked with a higher risk of AKI [33].

Using Lasso, we could identify some important variables associated with AKI non-survival patients and survival patients. The most important variable for Lasso in this study was the minimum creatinine (non-survivors: 1.07±1.19; survivors: 1.45±1.11, P<0.01). Other research showed the slope of the minimum creatinine (30.32%) was the most important variable for predicting AKI [34]. This indicates that the minimum creatinine was more useful in predicting AKI mortality than any of the other laboratory measurements or vital signs [34]. Since the study used only data available in the eICU-CRD, the result had some implications and require further research.

In this study, four machine learning methods (RF, LR, SVM, and XGBoost) were used to predict the mortality of AKI. Performance comparison results showed the XGBoost achieved the highest scores in AUROC, accuracy, and F1 score, and the second-highest score in recall and precision. XGBoost performed better than other machine learning models, and the advantages were statistically significant in AUROC and F1 score (P<0.01). While the XGBoost model has outstanding advantages, the XGBoost model has not been externally validated against other databases. Inconsistencies between different databases may limit the applicability and generalizability of the XGBoost prediction model, as each algorithm is limited by the quality of the data used for training and testing purposes. Although the clinical applicability of the XGBoost mortality prediction model still needs to be tested in actual clinical practice. However, due to its performance and clinical interpretability, we believe that the model may help clinicians avoid treatment delays in high-risk AKI patients. The XGBoost model can play an auxiliary role for clinicians in clinical decision-making.

Meanwhile, there were some limitations to this study. Firstly, although the data quality of the eICU-CRD database is high, the results obtained had certain limitations due to geographical limitations. For example, in this study, 76.6% of the included patients were Caucasian. The applicability of the predictive model to other populations or regions still requires external verification. Second, though the eICU database is considered tele-ICU data, the data collection mode and data source are not well defined. Third, the terminology variations across institutions and health information systems constitute additional obstacles [35]. The next step would be to explore the intrinsic relationships between features and further validate the model results using additional clinical data sets.

## Conclusions

The better prediction performance of XGBoost facilitates risk identification and early intervention of AKI patients at risk of death. It may be helpful to aid clinicians in making timely clinical intervention decisions for AKI patients, which is essential to help reduce the in-hospital mortality of AKI patients.
